# Tools for Evolutionary and Genetic Analysis (TEGA): A new platform for the management of molecular and environmental data

**DOI:** 10.1590/1678-4685-GMB-2018-0272

**Published:** 2020-05-29

**Authors:** Dario Ezequiel Elias, Eva Carolina Rueda Rueda

**Affiliations:** Universidad Nacional de Entre Ríos, Facultad de Ingeniería, Cátedra de Genética, Oro Verde, Entre Ríos, Argentina.; Universidad Nacional del Litoral, Facultad de Humanidades y Ciencias, Laboratorio de Genética, Ciudad Universitaria, Santa Fe, Argentina.; Consejo Nacional de Investigaciones Científicas y Técnicas (CONICET), Buenos Aires, Argentina.

**Keywords:** Bioinformatics, biological data management, population genetics, molecular markers, Open Science

## Abstract

Population genetics studies the distributions and changes in population allele frequencies in response to processes, such as mutation, natural selection, gene flow, and genetic drift. Researchers daily manage genetic, biological, and environmental data of the samples, storing them in text files or spreadsheets, which makes it difficult to maintain consistency and traceability. Here we present TEGA, a WEB-based stand-alone software developed for the easy analysis and management of population genetics data. It was designed to: 1) facilitate data management, 2) provide a way to execute the analysis procedures, and 3) supply a means to publish data, procedures, and results. TEGA is distributed under the GNU AGPL v3 license. The documentation, source code, and screenshots are available at https://github.com/darioelias/TEGA. In addition, we present Rabid Fish, the first implementation of TEGA in the Genetics Labortory of the Faculty of Humanities and Sciences at the National University of the Litoral, where research focuses on population genetics studies applied to non-model organisms.

## Introduction 

As a part of evolutionary biology, population genetics, deals with the study of genetic differences within and between populations (Charlesworth and Charlesworth, 2017). Later, in 1987, using molecular markers (first mitochondrial DNA and later nuclear markers), John Avise introduced the concept of Phylogeography as a way of explaining how historical geological, climatic, and ecological conditions influenced the current distribution of species and genetic lineages Subsequently, advances in laboratory (especially in DNA sequencing technologies) and computational methods that make better use of data made phylogeographic inferences more accurate (Avise, 1998). Phylogeography has experimented significant growth in areas such as conservation, because it helps in defining evolutionary significant units, and in the study of prioritization for areas of high value for conservation (Moritz, 1992; Crandall *et al.*, 1999; Frankham, 2010). 

Although mitochondrial DNA has been broadly used, microsatellite markers are still valuable tools in molecular ecological and phylogeographic studies (Jarne and Lagoda, 1996; Vignal *et al.*, 2002; Selkoe and Toonen, 2006). Recent developments in next-generation sequencing approaches have also revolutionized the development of molecular markers, allowing rapid discovery of thousands of potential microsatellite *loci* in the genome of model and non-model organisms (Ewers-Saucedo *et al.*, 2016; Vartia *et al.*, 2016; De Barba *et al.*, 2017; McKendrick *et al.*, 2017). Despite its long success in obtaining diploid robust genetic information, the analysis of a population genetics data set typically involves a variety of software packages, each of them with a different input data format (Coombs *et al.*, 2008). In addition, researchers daily need to manage genetic, biological, and environmental data of the samples, storing them in text files or spreadsheets, which makes it difficult to maintain consistency and traceability. Here is where TEGA comes in. It is a WEB platform developed for easy data population genetics analysis and data management. 

## Material and Methods

TEGA was initially built with JHipster. The Back-End was developed in JAVA with Spring Boot and PostgreSQL. The procedures for the genotype analysis were implemented in bash using R and Python libraries. The Front-End was developed in JavaScript with AngularJS and Bootstrap.

## Results

TEGA is a WEB-based stand-alone software (WEBbased platform) that aims at facilitating the daily workflow in research focused on population genetics and molecular ecology. The purpose of TEGA is to contribute to the autonomy of the research teams, by providing them with a means to manage, analyze, and make their data and results available. 

To use TEGA, the research teams must download and install the platform on their server. TEGA has a user manual with instructions for its installation and use. Once installed, team members will be able to import, manage, and analyze their data. When the work is finished, the data, results (tables and graphics), and procedures can be accessed by other professionals through the platform. 

## TEGA’s objectives

### Facilitating data management

**Figure 1:**
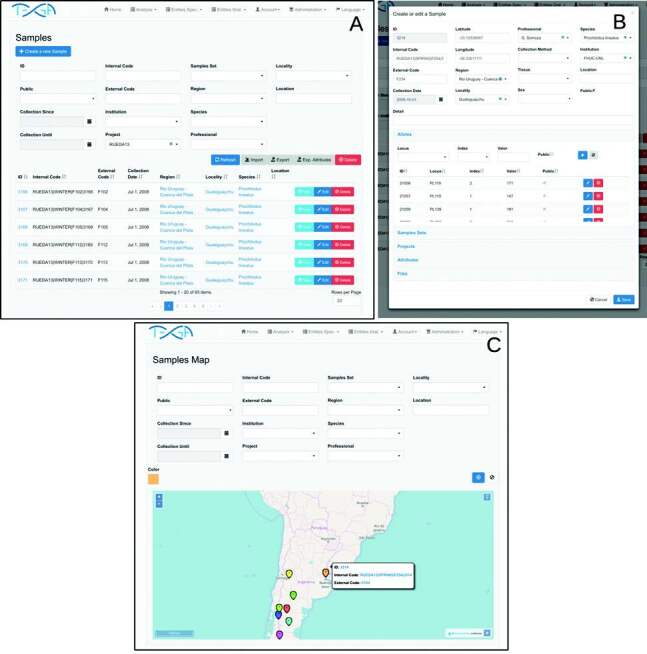
Sample screens. (A) Query screen; (B) edition and creation screen; (C) map screen.

TEGA has a structure based on entities to facilitate management. Every entity has screens with basic functions to: create, read, update, and delete (CRUD). It is also possible to bulk import sample and genotype data (*loci* and alleles). For some entities, like Samples, Projects, and Genotype Analysis, it is also possible to attach files (e.g., pictures and documents). Furthermore, given the large amount of data that can be linked to the samples, TEGA allows the user to create type-safe dynamic attributes and link them to different entities. In addition, it is possible to visualize the samples’ geographical position with OpenStreetMap (Figure 1). 

TEGA has also implemented a module for management and execution of data analysis procedures. A user with the Investigator role can create the procedures and attach the execution and configuration files, indicate the input data for the procedure (e.g., sample and allele data) and the parameters for execution. These procedures can then be executed from the Genotype Analysis screen. 

### Providing a way to execute the analysis procedures

**Figure 2:**
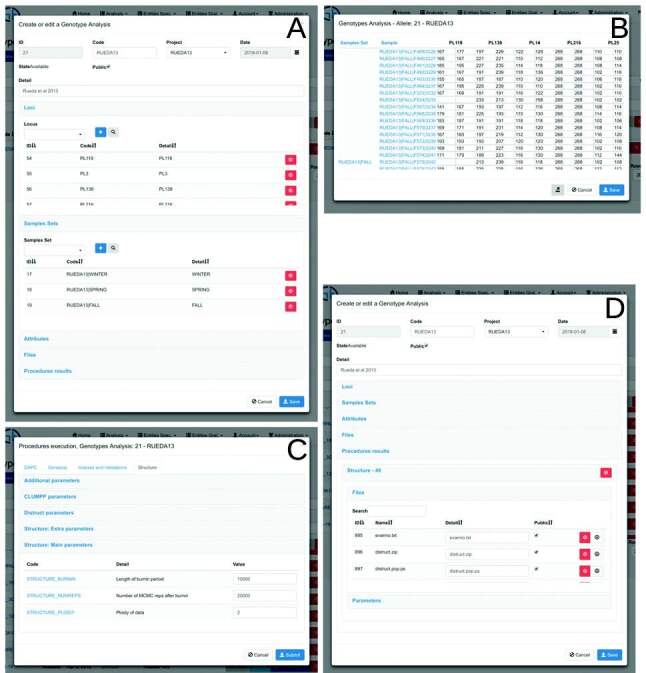
Genotypes Analysis screens. (A) Edition and creation screen (panel of *Loci* and Samples Sets); (B) query and edition screen of alleles included in the genotypes analysis; (C) screen of analysis procedures execution; (D) edition and creation screen (procedures results panel).

TEGA has an entity called Genotype Analysis for the management of data related to execution of genetic analysis procedures. Initially, the user must create sample sets that contain samples grouped according to a specific criterion (e.g., sampling sites or sampling date). Then, users must create a new genotype analysis, selecting the sample sets, *loci*, and the project linked to the analysis. It is then possible to execute the analysis procedures from the platform interface. Once a procedure is in execution, a genotype analysis cannot be edited or deleted, and when it finishes running, the user will get access to the result files from the analysis edition screen. In this way, TEGA links the procedure results with the entry data, procedure, and parameters used, facilitating traceability of the analysis (Figure 2). 

Although TEGA is designed so that users (members of the research team) can carry out their own analysis procedures, in its first version we implemented common methods for population genetics studies, like GENEPOP (Rousset, 2008), STRUCTURE (Pritchard *et al.*, 1998), and Discriminant Analysis of Principal Components (Jombart *et al.*, 2010) pipelines. TEGA includes empirical data to test them (Rueda *et al.*, 2013; Kamvar *et al.*, 2014). The user manual has the steps to do this in a very detailed way. 

### Supplying a means to publish data, procedures and results

TEGA has different user roles to allow private use of the data until the day of publication. Anonymous and Invited roles are intended for people outside the research team, who have read-only access to public data. Administrator and Researcher roles will only be for use by the investigation team members, who have access to public and private data, and can carry out CRUD operations and execute analysis procedures. 

When the results of a project are published, the users of the platform (with Researcher or Administrator roles) can switch the project status, changing it to public. This action will change the status of samples, alleles, *loci*, and the genotype analysis related with the project, in order to be explored by users with Anonymous or Invited roles. In this way, TEGA simplifies data and result publication. In addition, it is also possible to change the status of the analysis procedures. 

## Rabid Fish

Rabid Fish is the first TEGA implementation in the Genetics Laboratory of the Faculty of Humanities and Sciences at the National University of the Litoral (FHUCUNL). Its research goal is focused on population genetics studies, with emphasis in conservation questions related to non-model wetland organisms that are endangered or managed. The laboratory has obtained molecular markers (microsatellites) with traditional methods and NGS technologies (Rueda *et al.*, 2011a,b ; Metz *et al.*, 2016; Ojeda *et al.*, 2017). The fieldwork comprises the area of the La Plata basin, which it is the second-largest river basin of South America, including major rivers such as the Parana, Paraguay, and Uruguay systems. The research group obtained and analyzed fish samples from different migratory and commercially exploited species (Rueda *et al.*, 2011a,b, 2013, 2017; Metz *et al.*, 2016; Ojeda *et al.*, 2017) resulting in a huge collection of samples donated by many collaborators, with different biological issues, and including more than 20 sites from five countries and four species: *Prochilodus lineatus*, *Salminus brasiliensis*, *Leporinus obtusidens*, and *Pseudoplatystoma corruscans*. 

The implementation of TEGA in the Genetics Laboratory of FHUC-UNL simplified data management and allowed to publish the data and studies results, creating a new resource for the community, a database called Rabid Fish (see Internet Resources section). 

## Discussion

TEGA is a WEB-based platform developed for the easy analysis and management of population genetics data. There are WEB-based platforms with purposes similar to those of TEGA, for example DRIVERGENOME (Magalhães *et al.*, 2012) and LOVD (Fokkema *et al.*, 2011) (Table 1). LOVD focuses on the collection, display, and curation of DNA variants in *locus*-specific databases. The aim of DRIVERGENOME is to assist population genetics and genetic epidemiology studies performed by small-to-medium research groups, by providing storage, query, and format conversion functionalities. Both platforms focus on model organisms for which sequenced genomes are available, particularly *Homo sapiens*. Although these platforms offer by default tools to export genetic data in different formats and tools for the visualization of variants, they do not have tools to analyze, for example, the structure of the population. 

**Table 1:** Platforms for populations genetic data management.

	TEGA	DRIVERGENOME	LOVD
Aims	1) Facilitating population genetics data management.	Assist population genetics and genetic epidemiology studies performed by small-medium research groups, by providing storage, query, and format conversion functionalities.	Web-based software for the collection, display, and curation of DNA variants in locus-specific databases.
	2) Providing a way to execute the analysis procedures.		
	3) Supplying a means to publish data, procedures and results.		
Genetic data	STRs	SNPs, Indels, STRs and CNPs	SNPs, Indels, STRs and CNPs
Focused on a species	No. The species is an attribute of the samples.	Yes. *Homo sapiens*	Yes. *Homo sapiens*
Tools	Visualization:		
	- Samples Map		
		Data format conversion tools for population genetics software.	Data visualization tools and reference sequence parser.
	Analysis:		
	- STRUCTURE		
	- GENEPOP		
	- DAPC		
Extensibility	The user can add or modify analysis procedures without needing to modify the sources or restart the platform.	New conversion tools can be added by experienced users.	Developers can incorporate additional tools into the source code.
Dynamic Attributes	Yes	No	Yes
Reference	This study	Magalhães *et al.*	2012

STR = Short Tandem Repeats; SNP = Single Nucleotide Polymorphism; CNP = Copy Number Polymorphisms.

The main advantage of TEGA is its approach to the management and execution of the analysis procedures. TEGA allows to select the samples and *loci* for performing a genotype analysis, from where it is possible to execute multiple procedures. In addition, the user can append new procedures to the platform without having to modify its source code, or restarting it. As the procedures can be developed in any language, it is not necessary for the user to do this in the languages and frameworks used in TEGA. The intention is that the user can append the scripts he/she uses daily. In turn, the management also includes the results of the execution of the procedures, the indicated parameters, and the resulting files. In this way, TEGA carries out an integral management of the data, results, and procedures, which can then be published in the same way. We believe that this feature of TEGA will facilitate the access to data and procedures, and allow the easy reproduction of studies. We believe that this is aligned with the current needs of the scientific community, as reflected in the Open Data and Open Science movements (Fecher and Friesike, 2012; Nosek *et al.*, 2015). 

## Conclusion

TEGA is a WEB-based platform that aims at increasing the autonomy of researchers in the management, analysis and publication of data, procedures, and results. In the future, we plan to add other analysis tools, to integrate TEGA with other databases and improve their implementation and internationalization. 
